# Remote Monitoring Systems for Patients With Chronic Diseases in Primary Health Care: Systematic Review

**DOI:** 10.2196/28285

**Published:** 2021-12-21

**Authors:** Mariana Peyroteo, Inês Augusto Ferreira, Luís Brito Elvas, João Carlos Ferreira, Luís Velez Lapão

**Affiliations:** 1 NOVA School of Science and Technology NOVA University of Lisbon Setúbal Portugal; 2 Inov Inesc Inovação Instituto de Novas Tecnologias Lisbon Portugal; 3 Instituto Universitário de Lisboa (ISCTE-IUL) ISTAR Lisbon Portugal; 4 School of Biology St Leonard's Postgraduate College The University of St Andrews St Andrews United Kingdom; 5 Unidade de Investigação e Desenvolvimento em Engenharia Mecanica e Industrial Department of Mechanical and Industrial Engineering NOVA School of Science and Technology Setúbal Portugal; 6 Comprehensive Health Research Center NOVA Medical School NOVA University of Lisbon Lisboa Portugal

**Keywords:** sensors, wearables, remote monitoring, digital health, primary health care, chronic diseases

## Abstract

**Background:**

The digital age, with digital sensors, the Internet of Things (IoT), and big data tools, has opened new opportunities for improving the delivery of health care services, with remote monitoring systems playing a crucial role and improving access to patients. The versatility of these systems has been demonstrated during the current COVID-19 pandemic. Health remote monitoring systems (HRMS) present various advantages such as the reduction in patient load at hospitals and health centers. Patients that would most benefit from HRMS are those with chronic diseases, older adults, and patients that experience less severe symptoms recovering from SARS-CoV-2 viral infection.

**Objective:**

This paper aimed to perform a systematic review of the literature of HRMS in primary health care (PHC) settings, identifying the current status of the digitalization of health processes, remote data acquisition, and interactions between health care personnel and patients.

**Methods:**

A systematic literature review was conducted using PRISMA (Preferred Reporting Items for Systematic Reviews and Meta-Analysis) guidelines to identify articles that explored interventions with HRMS in patients with chronic diseases in the PHC setting.

**Results:**

The literature review yielded 123 publications, 18 of which met the predefined inclusion criteria. The selected articles highlighted that sensors and wearables are already being used in multiple scenarios related to chronic disease management at the PHC level. The studies focused mostly on patients with diabetes (9/26, 35%) and cardiovascular diseases (7/26, 27%). During the evaluation of the implementation of these interventions, the major difficulty that stood out was the integration of information into already existing systems in the PHC infrastructure and in changing working processes of PHC professionals (83%).

**Conclusions:**

The PHC context integrates multidisciplinary teams and patients with often complex, chronic pathologies. Despite the theoretical framework, objective identification of problems, and involvement of stakeholders in the design and implementation processes, these interventions mostly fail to scale up. Despite the inherent limitations of conducting a systematic literature review, the small number of studies in the PHC context is a relevant limitation. This study aimed to demonstrate the importance of matching technological development to the working PHC processes in interventions regarding the use of sensors and wearables for remote monitoring as a source of information for chronic disease management, so that information with clinical value is not lost along the way.

## Introduction

### Background

Digitalization of care processes, holistic sensing supported by the Internet of Things (IoT), and artificial intelligence (AI) tools are being actively applied with benefits to the health sector and giving rise to the smart health paradigm [1]**.** This corresponds to an emerging market that was evaluated at US $143.6 billion in 2019, with an estimated annual growth rate of 16.2% from 2020 to 2027 [2]. In this transformative process, health remote monitoring systems (HRMS) are recognized as an emerging technology that use sensors and wearable devices to collect patient data. However, to provide clinical value, these systems have to be associated with clinical processes and therapeutics so that measurements can be linked with actual patient care.

The use of sensors in health is a recent but growing area of research. These sensors are usually called biosensors, as they often collect patients’ vital signs. Wearable devices are devices that patients use in direct contact with the body to provide clinically relevant data for care. This continuous monitoring process defines personalized care. An IoT sensor can be used in either a discrete manner or a continuous manner [3]. A Scopus search showed that there were 96,888 publications regarding biosensors just in 2020.

Wearable devices are equipped with sensing capabilities for user mobility tracking and monitoring physical activity (for example, counting steps), heart rate, oxygen levels, and blood pressure. For instance, a wearable electrocardiogram monitor has been used by many patients with serious conditions under the prescription of their physicians [4]**.** Another example of a wearable device used by patients is a wearable blood pressure monitor, which can be integrated into a watch [5]. There is, however, the need for proper evaluation or certification to regulate the efficacy of these wearables, as some frauds have already been identified. The Food and Drug Association in the United States has established a wearables certification [6]**.** Lastly, there are also patch biosensors, which are self-adhesive patches that can collect a range of different data such as heart rate, respiratory rate, temperature, and body posture and can detect falls.

The implementation of HRMS is based on an IoT system that incorporates, stores, and communicates the information gathered by a set of wearable devices and sensors. The computer senses and records the daily physiological data of the patient by means of a data processing device, data transition, data archive, data analytics, and AI [7]. An HRMS is based on 4 main pillars: (1) development of systems to identify disease progression and prevention through remote sensors; (2) use of big data (BD) processing and analysis, which processes multiple heterogeneous data sources to integrate different patient data, aiming at providing high-quality personalized treatment; (3) development of predictive models supported by AI to be implemented on top of the processed BD, allowing the classification of patients and the discovery of behavioral patterns to enable alerts to be generated when an abnormality is registered for quicker clinical action; and (4) create an entirely remote interaction process from the hospital to the patient. The HRMS enables a data-intensive approach, in which a large amount of health data is generated, stored, and available for data mining, allowing the generation of useful knowledge. All data processed from IoT sensors and wearable devices can be input into BD analytics, which allows the generation of knowledge and alerts that can be used to monitor health [8].

The use of HRMS to record data supports the development of personal health records, involving patients in their own data collection, health monitoring, exercise, and lifestyle [9]. A single health database allows personalized care, as the health care professional can tailor the treatment according to both the patient conditions and the device readings. The term personalized care refers to the design and adaptation of clinical treatment to the characteristics, needs, and individual preferences of the patient throughout all stages: care, prevention, diagnosis, treatment, and follow-up [10]. The use of this term has been growing in recent years, as more recent technology such as genome sequencing, wearable devices, and HRMS has allowed the use of precision medicine [11]. The area of personalized medicine has rapidly grown over the last decade due to improvements in areas such as diagnostic testing, BD technologies, and others. The European Union is highly concerned about the provision of high-quality personalized medicine [12].

Communication of devices with cloud platforms allows for data to be stored in the cloud and easily accessed by doctors, allowing remote health monitoring functions. Remote patient monitoring requires machinery that collects and interprets biometric and physiological data [13]. Remote patient monitoring has many applications such as real-time illness detection, continuous monitoring of patients such as those with chronic disease or less severe conditions, or monitoring of athletes’ health [13]. Recent guidelines have pointed out that the creation and adoption of person-centered integrated care for older adults is critical, as a decline in intrinsic capacity is reported among older adults. Intrinsic capacity includes the mental and physical attributes that the patient can use to perform any daily task, can be used to identify those patients who would benefit more from interventions, and can be measured by wearable devices [14].

### Chronic Diseases in the Primary Health Care Context

Chronic diseases are among the most important health problems to benefit from HRMS. The role of primary health care (PHC) centers as the first point of contact is considered a universal health coverage model. For that reason, health care data management and remote monitoring benefit from being included in PHC, enabling a comprehensive collection of data from patients and making the data available to health professionals afterwards.

Not only has research into wearable medical devices increased but also the availability of these devices to the general public. Devices such as mobile phones and smart bands, usage of which is becoming customary, are increasing the amount of data generated that can be used to improve the management of chronic diseases. More than 100,000 apps have been created to use this type of data, and the number has been doubling every 2.5 years [15]. However, often these applications cannot be integrated into the health care process, resulting in dispersed data.

PHC is suffering from larger demand from an increase in the number of patients with chronic diseases. Due to hospital overload, the use of remote monitoring systems is often considered of added value. Systematic monitoring would also allow for remote tracking of symptom progression in less severe COVID-19 patients, allowing one to closely monitor and increase the comfort of patients, but also reduce the strain on the health system. An HRMS is fundamental for supporting the collection of data necessary to improve the management of novel conditions, such as COVID-19, as it allows the collection of data useful in medical research and to identify patterns of symptoms that could indicate patients’ symptom progression [16].

Furthermore, remote monitoring systems might improve health professionals’ effectiveness in managing chronic diseases, as an HRMS permits the early detection of disease warning signs, which is crucial to improve survival rates of specific diseases (eg, hypertension, diabetes, chronic obstructive pulmonary disease). These systems promise to, by tracking patients’ disease progression, increase patients’ awareness of and engagement with chronic therapeutics.

### Goal of the Study

Although several other studies are being conducted on this topic, it is paramount to analyze published studies (beyond clinical trials or pilot studies) to understand the weaknesses and opportunities that still persist in this area

This paper aimed at performing a systematic review of the literature on HRMS in the PHC setting, identifying the current status of the digitalization of the health process, regarding (1) digital monitoring of chronically ill patients, (2) early detection of acute episodes in patients with decompensated chronic pathologies, (3) the outcomes of the implementation process, and (4) patient empowerment.

As secondary outcomes, the following was also assessed: (1) the digital communication between PHC professionals and patients or caregivers, (2) the integration of the information collected by the health care information systems, (3) reduction of hospital burden, and (4) user satisfaction (patient, caregivers, and professionals).

## Methods

### Search Strategy and Inclusion Criteria

A systematic literature review was conducted by following PRISMA (Preferred Reporting Items for Systematic Reviews and Meta-Analysis) methodology [17] and with the research question: “What is the state of the art on healthcare remote monitoring system usage for chronic patients in primary health care?”

We searched the databases of Scopus and Web of Science Core Collection (WoSCC), and the research was conducted through December 31, 2020. The results had to be articles, published between 2015 and 2020, and written in English or Portuguese. The documents collected were only about computer science, medicine, engineering, and health professions.

The search strategy was based on 6 queries, each with a different focus of research (detailed in Multimedia Appendix 1). This method allowed for the observation of the number of articles existing in both databases, considering the concept and context as well as the population under study. It is important to note that the values corresponding to the queries still have duplicate articles.

For this review, only articles were considered. Grey literature, reviews, conference papers, workshops, books, and editorials, as well as works not related to the domain, were excluded. The population included all ages, genders, and ethnic groups diagnosed with multimorbidity or at least one chronic disease. The study was considered eligible for inclusion if the intervention included one of these criteria: (1) continuous electronic recording of patient indicators (sensors or wearables) connected to a computer system integrated into PHC centers, (2) patient input devices linked to a computer system allowing the display of data in real time for analysis by PHC professionals, and (3) collection of personal electronic health or clinical data transmitted for review by a remote PHC professional.

### Study Selection

The initial selection of papers was done using the title and abstract, and in some cases in which that information was insufficient, the full document was analyzed. The process was performed by 3 researchers independently: 2 performed the process, and in case of disagreement, the third resolved the disagreement.

### Data Extraction and Synthesis

The data were managed and stored by Zotero and Microsoft Excel version 16.46 (21021202). These data were title, author, year, journal, subject area, keywords, and abstract. For data synthesis and analysis, a qualitative assessment was conducted based on the results presented in the previous section. The databases—Scopus and WoSCC—were searched systematically regarding the published work related with the concept “Healthcare Remote Monitoring Systems” or “Smart Health,” with the target population “Chronic Patients,” and within the context of the study “Primary Health Care.”

## Results

### Queries and Themes

**Figure 1:**
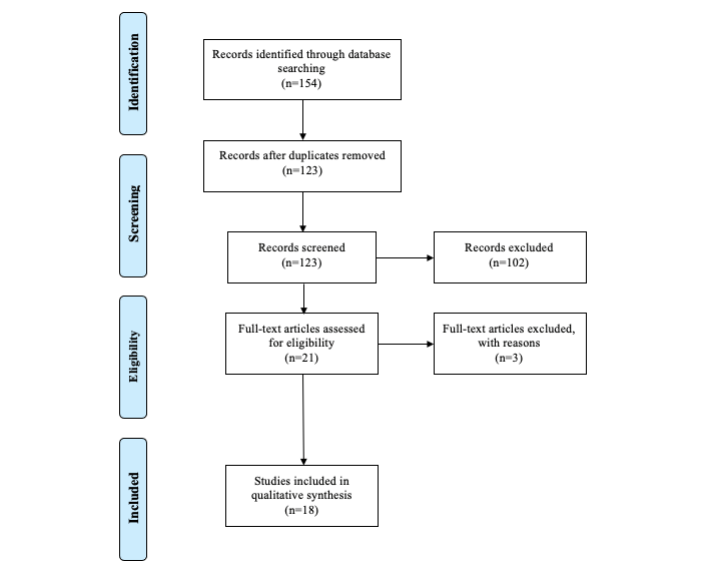
PRISMA (Preferred Reporting Items for Systematic Reviews and Meta-Analysis) workflow diagram.

The research was conducted using queries and themes. Each query was conducted in the individual databases and with the same restrictions and filters. Figure 1 shows the PRISMA workflow diagram from the total number of articles studied.

With query 1, Scopus and WoSCC were searched for literature regarding the concept of this study, and we found 61,230 results.

With query 2, the search included the target population of patients with a chronic disease, and 61,469 documents were found in both databases.

With query 3, which involved the context of this study, 73,550 documents were found in Scopus and WoSCC.

With the fourth query, the databases were searched for the concept of the study and the target population but without any context, and 1458 documents were found.

With query 5, queries 1, 2, and 3 were combined, and the documents collected involved all the aforementioned inclusion criteria. The merging of all the queries resulted in 154 documents. After performing a manual process to identify significant subjects based on the research questions, identify the outcomes, and remove the duplicates, 18 documents were obtained. Our systematic research took into account year, area, research question topic, and a short description. We also looked at articles published in 2020 for the COVID-19 pandemic effect.

Regarding these results, additional research was conducted (query 6) with which the purpose was to compare the results in both databases for HRMS used with patients with a chronic disease but in another specific context (in hospital care). In the search, query 1 and query 2 were combined, and hospital care was added (“Hospital” OR “Acute Care” OR “Clinical”), resulting in 726 results.

### Study Characteristics

All 18 studies included in the review were selected through the use of the aforementioned specific criteria. Table 1 shows the key study characteristics regarding the year, region, disease of focus, interface, data collection methods, collection frequency, stakeholders’ involvement, and existence of pilot studies. Classification of the studies regarding these characteristics was not mutually exclusive, given that these were assigned due to presence or absence in the study.

**Table 1:** Study characteristics (n=18).

Characteristics		Articles, n (%)
**Region**		
	Europe	8 (44)
	Americas	7 (39)
	Southeast Asia	1 (6)
	Africa	1 (6)
	Western Pacific	1 (6)
**Disease of focus (n=26)**		
	Diabetes	9 (35)
	Cardiovascular diseases	7 (27)
	Respiratory diseases	4 (15)
	Multimorbidity	4 (15)
	Mental disorders	2 (8)
**Interface (n=20)**		
	Mobile phone or telephone	10 (50)
	Tablet	6 (30)
	Web-based platform	4 (20)
**Data collection method (n=19)**		
	Sensors	9 (47)
	Questionnaires	5 (26)
	Wearables	5 (26)
**Collection frequency (n=14)**		
	Daily	6 (43)
	Monthly	4 (29)
	Weekly	3 (21)
	Permanent	1 (7)
**All stakeholders involved**		
	Yes	9 (83)
	No	6 (33)
**Pilot study**		
	Yes	8 (44)
	Being developed	3 (17)
	No	7 (39)

### Outcomes Analysis

The results previously defined in the goal of this review are summarized in Table 2. The description of the indicators was explicit, and no requests to the authors of the articles for clarification were necessary. As mentioned before, classification of the studies regarding the outcome was not mutually exclusive, given that these were attributed due to presence or absence in the study.

**Table 2:** Outcome comparison.

Outcomes		References	Number of documents
**Primary outcomes**			
	Digital monitoring of the patients’ chronic diseases	[18-35]	18
	Early detection of acute episodes	[18-24,27-29,31-35]	15
	Outcomes of the implementation studies: effectiveness and cost-effectiveness	[18,19,22,28,30,33,34]	7
	Outcomes of the implementation studies: implementation process with multidisciplinary teams	[18-21,23,24,27-31,33]	12
	Patient empowerment (self-management applications)	[18,19]	2
**Secondary outcomes**			
	Digital communication between PHC^a^ professionals and patients	[18-32,34]	16
	Integration of information into PHC centers	[18,20,26]	3
	Reduction of hospital inflow	[18-21,23-29,31,33-35]	16
	User satisfaction (patients, carers, and professionals); role of informal carers, especially to facilitate the use of technology by older adult patients	[19,21,28]	3

^a^PHC: primary health care.

### Risk of Bias in Included Studies

Given the categorization of articles included in the study, there may be bias in the definition of “Remote Patient Monitoring.” Thus, it is possible that some articles may have been excluded.

## Discussion

### Principal Findings

After assessing all the included studies, it was possible to acknowledge the growing prevalence of remote monitoring systems worldwide in recent years. Although the main goals across the different studies varied, the majority of the articles included common features for these devices. The interventions included the following features: medical condition management (n=3), diagnosis (n=1), conceptual models (n=6), reminders and alerts (n=2), self-reported monitoring (n=5), wearable remote monitoring device (n=4), health promotion and education (n=4).

Most of the articles selected included research about conceptual models regarding work methodologies or efficacy of the devices or the interventions. Of the articles, 24% (4/18), which had a specific focus on wearable remote monitoring systems for patients with a chronic disease in PHC, were studies or proposals for conceptual care models. The topics in these studies varied from changes in chronic disease management through the use of biosensors to the impact assessment of the implementation of telecare projects. From these studies, approximately 75% (3/4) were theoretical models that focused on the understanding of barriers and challenges to digital transformation, but no context-related implementation evidence was presented to support the conclusions drawn.

The research using different queries with different keywords allowed the comparison of the state of the art regarding the concept in the chronic care context. Therefore, it was possible to observe the difference in HRMS applications for patients with a chronic disease carried out in both the hospital and PHC contexts. Given that chronic disease management is a major focus of PHC services [36], it would be expected that most studies would be applied in that setting.

However, by observing and evaluating the number of articles resulting from the research between contexts (see query 5 vs query 6), it was possible to verify that the influx of studies in the hospital context (n=726) was 7 times higher than studies in the PHC context (n=100). This difference may be due to the recent restructuring (since 2014) of health service contexts or to the difficulty in implementing studies in the PHC context [37]. This can also be explained by the fact that there are many clinical conditions that are still managed from the hospital point of view that focus on symptomatic treatment.

According to World Health Statistics 2020, it was estimated that 71% of deaths worldwide were caused by noncommunicable diseases (cardiovascular diseases, cancer, chronic respiratory diseases, and diabetes) [38]. In this literature review, it was possible to observe that the use of smart health is mostly focused on noncommunicable diseases that not only cause most deaths but also can be better controlled and prevented by controlling risk factors and monitoring of the patient’s health [39]—see the “Disease of focus” information in Table 1.

### Remote Monitoring and the COVID-19 Pandemic

With the COVID-19 pandemic, the role and importance of health digitalization had a special focus for the measures implemented to reduce the risk of transmission, not only because of the extreme need for adaptation of the entire population to the situation and the social and economic impact that this pandemic brought but also because of the pressure and exhaustion of health professionals (eg, burnout).

It was possible to identify 4 articles [21,25,33,34] in which the focus and role of health digitalization, through telecare, were seen as essential and urgent for the current or future pandemics and crisis situation management. The small number of articles found may suggest the need for further research and studies, to assess the reach of COVID-19 not only in other countries, particularly those where mental health infrastructures are less developed [40], but also in other vulnerable populations (eg, children, adolescents, older adults) and in areas facing barriers to accessing health care [41].

As presented in the Results section, 67% (12/18) of the studies included in this literature review presented, in some way, an implementation process (ie, proof of concept, pilot study, or clinical trial). However, it is necessary to take into account that, in 28% (5/18) of the studies in which proposals, evaluations, or conceptual models were presented, real and scientific evidence of the study success and effectiveness was still absent.

Most studies presented as a limitation the difficulties of large-scale implementation, often due to lack of clinical context, which could be improved by the participation of health workers and patients in the design and implementation process.

Some articles that have already conducted a proof of concept in pilot studies considered extrapolating the use of remote monitoring systems to many patients simultaneously as a limitation. Furthermore, those who focused on project evaluations mentioned that the biggest weakness was large-scale implementation, not only for stakeholders but also for the system users themselves. Still, the articles that focused on evaluations were, as already mentioned, only conceptual models that still lacked proof and real-world evidence.

Despite all the studies and results analyzed, there is still a dearth of scientific consistency in the development and implementation process of these interventions and devices [42,43]. According to Gagnon et al [44], the success of an intervention linked to telecare lies in the implementation process. Thus, and as it can be observed in this systematic literature review, it is possible to confirm that the weaknesses mentioned as limitations in the reviewed articles are included in the critical success factors already described by the MOMENTUM framework [45].

Failures include the low adherence by users and, in particular, health professionals. The justifications for the failure of remote monitoring include the difficulty of integrating into existing working methods [23,27], the limited integration of data and information provided in existing communication systems [24,28,29,31], and the lack of correlation between the results of these interventions and specific and individual clinical knowledge for patients with a chronic disease [46]. There is a need for better methodological and evaluative approaches to the development and evaluation of health care improvement interventions [47].

### Comparison With Prior Work

Systematic reviews have been based on studies of the (1) identification of opportunities and barriers and (2) acceptability, effectiveness, and impact of the development and implementation of new methods of chronic disease management using remote monitoring systems (eHealth, mobile health, and telehealth). As stated by Trifan et al [46], many reviews that have already been conducted focused on specific conditions and pathologies or had a general focus without a clinical context, as can be seen with this study. However, only a few review studies considered the use of sensors and wearables as a method to collect information for remote monitoring [48-57].

This study aimed to demonstrate the importance that integration and contextualization have in the development and implementation of interventions regarding the use of sensors and wearables for remote monitoring as a source of information for chronic disease management in PHC. To avoid losing information with clinical value throughout the process, it is necessary for not only technological development to be adequate and innovative but also human resources and work methodologies to be able to adapt to new changes.

The PHC context integrates multidisciplinary teams and patients with often complex chronic pathologies. As such, the implementation of new methods and processes for chronic disease management has to be phased and patient-centered and involve all stakeholders [45]. The impact on health care working processes is still not very well studied.

The technological evolution has enabled remote monitoring to grow almost exponentially [56], to solutions, artefacts, or devices that are increasingly smaller, faster, and easier to use and ready to be integrated into the clinical context. The integration of these devices could allow for cost reduction, improved patient quality of life, and early detection of acute episodes, enabling more adequate and personalized intervention and management of disease according to the needs of each patient [57]. However, integrating them in the context of care, specifically in PHC, means that the developed devices have to be adapted not only to patients’ specific needs but also to health care professionals’ requirements. With the involvement of health care professionals, it is possible to design a solution that takes into account not only the technological requirements behind the system but also the medical requirements, thereby contributing to the improvement of disease management for patients with complex pathologies such as chronic diseases [58].

### Limitations

The limitations identified in this systematic review are the use of only 2 databases and the exclusion criteria, which may have led to the exclusion of relevant articles, as well as the time between the search and review process. In addition, most studies were conducted in a hospital setting, and only a few were conducted in a PHC setting; therefore, another limitation is the low number of studies in a PHC context.

### Conclusions

This literature review identified several studies on the implementation of remote monitoring devices for patients with chronic diseases in the PHC context. These studies were mainly of cardiac, respiratory, or metabolic pathologies. Despite the opportunities observed, the limitations presented are based on difficulties in generalizing the studies and implementing them on a larger scale. This may be due to both the lack of senior managerial engagement as well as the lack of contextualization of the solutions presented, which, despite being able to prove the technology concept, are not compatible with the health professionals’ working methods or with the complexity required for multimorbid patients.

It is clear that innovative technological solutions are being developed. In order to fulfil the need in the area, these technologies have to be properly selected and adapted to the context of the patients as well as to the health care environment, meaning that more research will be necessary to improve knowledge in this field.

## Appendix

Multimedia Appendix 1 Detailed search strategy.

## Abbreviations

AI: artificial intelligence
BD: big data
HRMS: health remote monitoring systems
IoT: Internet of Things
PHC: primary health care
PRISMA: Preferred Reporting Items for Systematic Reviews and Meta-Analysis
WoSCC: Web of Science Core Collection
