# Supplementary material for: Remote Monitoring Systems for Patients With Chronic Diseases in Primary Health Care: Systematic Review
Source: JMIR Mhealth Uhealth. 2021 Dec 21;9(12):e28285. doi: 10.2196/28285 (PMC8734917; doi:10.2196/28285)
Supplement: Multimedia Appendix 1 [file mhealth_v9i12e28285_app1.docx]

Multimedia Appendix 1. Detailed Research Strategy

**Main Query:**

**Query 1** – (“*Remote Monitoring*” OR “*Digital Monitoring*” OR “*Wearable**” OR “*Biosensor**” OR “*Smart Health*” OR “*eHealth*” OR “*Telemedicine*”) AND (“*Chronic Disease**” OR “*Chronic Patient*” OR “*Multimorbidity*”) AND (“*Primary Health Care*” OR “*Primary Care*”)

**Additional Queries** (*not analysed, just to comparison*)**:**

**Query 2** – (“*Remote Monitoring*” OR “*Digital Monitoring*” OR “*Wearable**” OR “*Biosensor**” OR “*Smart Health*” OR “*eHealth*” OR “*Telemedicine*”)

**Query 3** – (“*Chronic Disease**” OR “*Chronic Patient*” OR “*Multimorbidity*”)

**Query 4** – (“*Primary Health Care*” OR “*Primary Care*”)

**Query 5** – (“*Remote Monitoring*” OR “*Digital Monitoring*” OR “*Wearable**” OR “*Biosensor**” OR “*Smart Health*” OR “*eHealth*” OR “*Telemedicine*”) AND (“*Chronic Disease**” OR “*Chronic Patient*” OR “*Multimorbidity*”)

**Query 6** – (“*Remote Monitoring*” OR “*Digital Monitoring*” OR “*Wearable**” OR “*Biosensor**” OR “*Smart Health*” OR “*eHealth*” OR “*Telemedicine*”) AND (“*Chronic Disease**” OR “*Chronic Patient*” OR “*Multimorbidity*”) AND (“*Hospital*” OR “*Clinical*” OR “*Acute Care*”)
